# The capability of deep-radiomics to predict pathological response to neoadjuvant immunochemotherapy in non–small cell lung cancer: a retrospective multicenter study

**DOI:** 10.3389/fimmu.2026.1770042

**Published:** 2026-02-27

**Authors:** Yuanxin Ye, Yuchi Tian, Lingling Wang, Zihan Xi, Yangfan Zhang, Tong Zhou, Zhenhua Zhao, Yifeng Zheng, Xiaoyun Liang, Haitao Jiang

**Affiliations:** 1 Department of Radiology, Zhejiang Cancer Hospital, Hangzhou, China; 2 Institute of Research and Clinical Innovations, Neusoft Medical Systems Co., Ltd, Shanghai, China; 3 Department of Radiology, Huzhou Central Hospital, Huzhou, China; 4 Department of Radiology, Shaoxing People’s Hospital, Shaoxing, China

**Keywords:** deep learning, delta-radiomics, neoadjuvant immunochemotherapy, NSCLC, pathological complete response

## Abstract

**Background:**

To establish a predictive model that combines radiomics, deep learning and clinical features for predicting the pathological complete response (pCR) of non-small cell lung cancer (NSCLC) patients after neoadjuvant immunochemotherapy (NIT).

**Methods:**

We retrospectively collected patients from three centers (split into training, internal testing and external testing cohorts). In this study, tumor segmentation was performed on chest CT images before (pre-NIT) and after (post-NIT) neoadjuvant therapy. The radiomics features were extracted from pre-NIT and post-NIT images. Deep learning (DL) features were extracted from the post-NIT images. The most meaningful features were selected using the mRMR and LASSO. A logistic regression classifier was then applied to create a classification model to predict pCR or non-pCR. The predicted probabilities were referred to as the Rad-scores and Deep-scores. Finally, Rad-scores, Deep-scores, and meaningful clinical features were fused to build a combined model.

**Results:**

A total of 178 patients were enrolled in the current study. In conventional radiomics, the efficacy of post-NIT model was better than the pre-NIT. In delta radiomics model, delta1 had the best efficacy. Subsequently, the post-NIT and delta1 features were further constructed as the combined model 1 with AUCs of 0.939 and 0.849, respectively. iRECIST was combined with the radiomics and the DL features to establish the combined model 2, which achieved the best performance among all the models, with AUCs of 0.955(training), 0.882(In-testing), and 0.839(Ex-testing).

**Conclusions:**

Our results demonstrated that combination of three dimensional features can provide complementary information to predict pCR more accurately.

## 1 Background

Lung cancer is the second most commonly diagnosed malignancy worldwide and remains the leading cause of cancer-related deaths (1). Non–small cell lung cancer (NSCLC) accounts for approximately 80% of all lung cancer cases. Neoadjuvant immunochemotherapy (NIT), combining immune checkpoint inhibitors (ICIs) with chemotherapy prior to surgery, has emerged as a promising strategy to enhance survival outcomes in NSCLC patients (2). Accurately predicting the efficacy of NIT before surgery holds significant clinical value, as it can reduce unnecessary exposure to potentially ineffective treatments, preventing delays in administering alternative therapies. Despite the promising potential of NIT, predicting individual patient response remains a clinical challenge, as traditional radiologic assessments based on tumor size changes may not accurately reflect therapeutic efficacy (3). Although, some microscopic biomarkers such as tumor mutation burden (4) and programmed death-ligand 1 (PD-L1) expression (5) have been associated with responses to ICIs, obtaining a comprehensive evaluation of tumor status through biopsy is limited by spatial and temporal tumor heterogeneity (6). Pathologic complete response (pCR) has been recognized as a surrogate endpoint for long-term survival outcomes (2, 7–9). Notably, patients achieving pCR in trials like NADIM (8) and CheckMate 816 (2) showed significantly enhanced event-free survival. Currently, pCR status can only be determined postoperatively through histopathological examination, underscoring the need for a non-invasive, preoperative predictive method.

To address this need, non-invasive imaging techniques and advanced analytical methods have been being explored as potential solutions for preoperative prediction of pCR status. Medical imaging provides a wealth of quantitative data that can serve as potential biomarkers (10). Radiomics, the extraction of high-dimensional features from medical images, has been utilized to assess tumor diagnosis and treatment (11–14). Delta-radiomics, which analyzes the temporal changes in radiomics features across multiple time points, offers a non-invasive approach to capture treatment-induced alterations in tumors (15). Meanwhile, deep learning techniques (16, 17), particularly convolutional neural networks (CNNs), have demonstrated exceptional ability to automatically learn and extract complex patterns from medical images, surpassing traditional feature-based methods in various predictive tasks (18, 19). To date, no studies have investigated the predictive value of models that integrate delta-radiomics and deep learning features for preoperative prediction of pCR in NSCLC patients undergoing NIT.

Therefore, the aim of this study is to develop and validate a novel predictive model that combines delta-radiomics, deep learning, and clinical features to non-invasively predict pCR in NSCLC patients receiving NIT. By leveraging the complementary strengths of these approaches, we seek to provide a cost-effective and repeatable tool to aid in treatment planning and improve patient outcomes.

## 2 Methods

### 2.1 Population and study design

**Figure 1:**
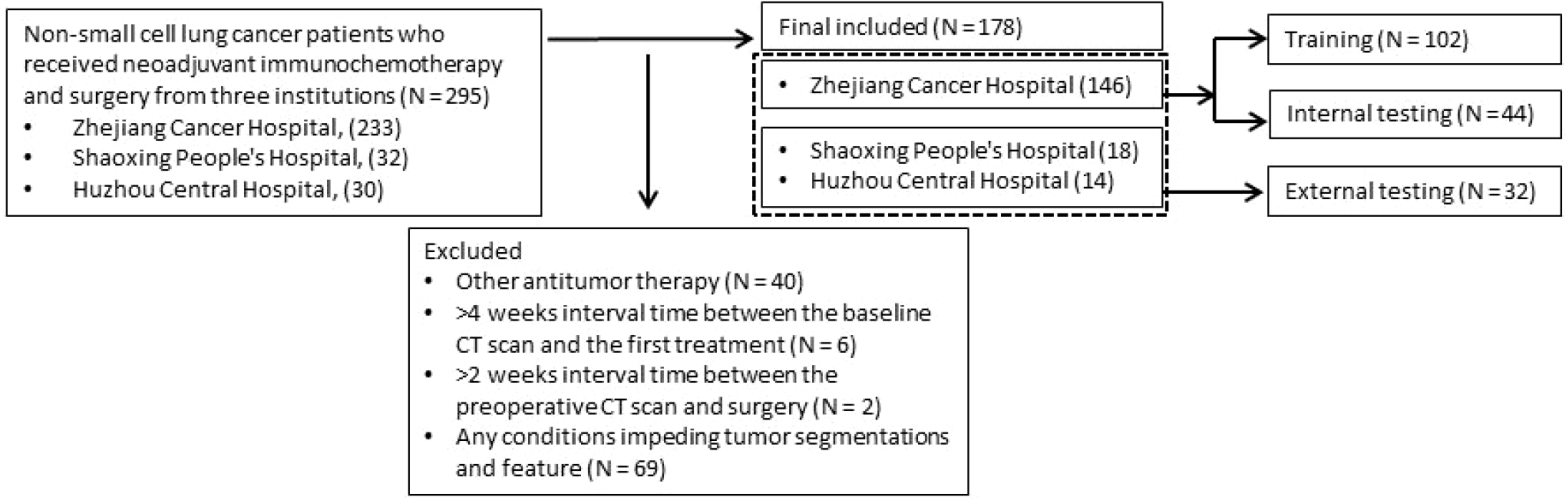
The flowchart for patient selection.

The patient selection and distribution flowchart is shown in **Figure 1**. NSCLC patients from three institutions (Zhejiang Cancer Hospital, from May 2018 to March 2021; Shaoxing People’s Hospital, from August 2019 to October 2021; Huzhou Central Hospital, from March 2020 to November 2022) who received NIT and surgery, were retrospectively enrolled. Criteria for inclusion: (a) receiving 2–4 cycles of NIT, (b) contrast-enhanced CT before NIT (baseline CT), (c) contrast-enhanced CT after NIT (preoperative CT), and (d) pathologically confirmed NSCLC, with pathological response described in the postoperative pathology report. Criteria for exclusion: (a) history of a different antitumor therapy, (b) >4 weeks interval time between the baseline CT scan and the first treatment, (c) >2 weeks interval time between the preoperative CT scan and surgery; or (d) any conditions impeding tumor segmentations and feature extraction (e.g., obvious image artifacts or lesions showing imaging complete response post-NIT).

The patients from Zhejiang Cancer Hospital were randomly assigned to the training and internal testing cohorts, with a ratio of 7:3. Patients from other two institutions were integrated as the external testing cohort. This study was reviewed and approved by the Ethics Committee of Zhejiang Cancer Hospital (IRB-2022-710), and the requirement for informed consent was waived.

### 2.2 Data collection

#### 2.2.1 Pathologic complete response

Pathological responses were assessed by two pathologists with >10 years of working experience according to the international guidelines (20). The definition of pCR was the complete lack of detectable viable tumor cells following a comprehensive assessment of the lung tumor and lymph nodes. The endpoint of this study was whether pCR was achieved, according to which patients were divided into pCR and non-pCR groups.

#### 2.2.2 CT imaging acquisition

CT images scanned on two time points (before and after NIT) need to be collected. CT scans were conducted on GE Healthcare Optima CT680 Series, Siemens Somatom Definition Flash, and Philips Ingenuity CT. The patients were scanned ranging from the thoracic inlet down to the costophrenic angle. Scanning protocols were as follows: 120-kVp, automatic tube current, and 512 × 512 matrix. Patients underwent contrast-enhanced chest CT using 100 mL of nonionic contrast media with an injection flow rate of 3 mL/s. Enhanced CT scanning began with data acquisition 50–60 s after contrast injection with the reconstructed layer of 1 mm thick and 1 mm apart.

#### 2.2.3 Clinical data

Clinical data were reviewed from medical records. Clinical data included the ICI type, age, number of treatment cycles, sex, smoking, pathological type, clinical staging, serological markers (cytokeratin 19 fragment [CYFRA21-1], neuron-specific enolase [NSE], cancer antigen 125 [CA125], cancer antigen 199 [CA199], carcinoembryonic antigen [CEA], and squamous cell carcinoma antigen [SCC]), and immune response evaluation criteria in solid tumors (iRECIST) profiles.

### 2.3 Tumor region segmentation

Two time-point CT images in DICOM format were exported from the Picture Archiving and Communication Systems, encompassing non-contrast lung window images and contrast-enhanced mediastinal window images. Subsequently, those CT images were imported into 3D Slicer to segment the volume of interest (VOI) by two radiologists with over ten years of experience in diagnosing thoracic lesions. If multiple lesions were present, the largest one was chosen as the target lesion. The radiologist performed a semi-automatic delineation of the target lesion on the lung window (**Supplementary Methods Part 1**). To ensure the consistency and accuracy, a total of 30 cases were randomly selected for re-delineation after three months. The consistency of the extracted features was assessed utilizing the intraclass correlation coefficient (ICC), where an ICC value exceeding 0.75 was indicative of strong consistency.

### 2.4 Radiomics feature extraction

Radiomics features of the VOI were extracted before and after neoadjuvant therapy using the PyRadiomics package in Python (version 3.6). These features were referred to as pre- and post-radiomics feature datasets, respectively. Each dataset consisted of 1,037 radiomics features (See **Supplementary Methods Part 2** for details).Subsequently, different delta-radiomics features were defined as follows:

1. Delta 1: pre-post, representing the absolute change in radiomics features before and after neoadjuvant therapy.
2. Delta 2: (pre-post)/pre, representing the percentage change in radiomics features before and after neoadjuvant therapy.
3. Delta 3: (pre-post)/(pre * number of immunotherapy cycles), representing the average percentage change per therapy cycle in radiomics features before and after neoadjuvant therapy.

### 2.5 Deep learning feature extraction

We employed a pre-trained 3D ResNet-18 model (21) to extract deep learning features from imaging data. The inputs to the network were segmented ROI volumes (masked tumor regions) rather than whole slices. Each ROI was cropped from the original image based on its bounding box and then resized to a uniform dimension of 128×128×32. The deep learning features were obtained from the activations of the final global average pooling layer, yielding a 512-dimensional feature vector for each sample. No data augmentation was applied during the fine-tuning process to maintain the integrity of the clinical imaging patterns and ensure the stability of feature extraction. During the fine-tuning process, the model utilized a binary cross-entropy loss function and the Adam optimizer, with an initial learning rate of 0.001 and a batch size of 8. To maintain the integrity of the clinical imaging patterns and the stability of feature extraction, no data augmentation was applied.

### 2.6 Feature selection and model building

**Figure 2:**
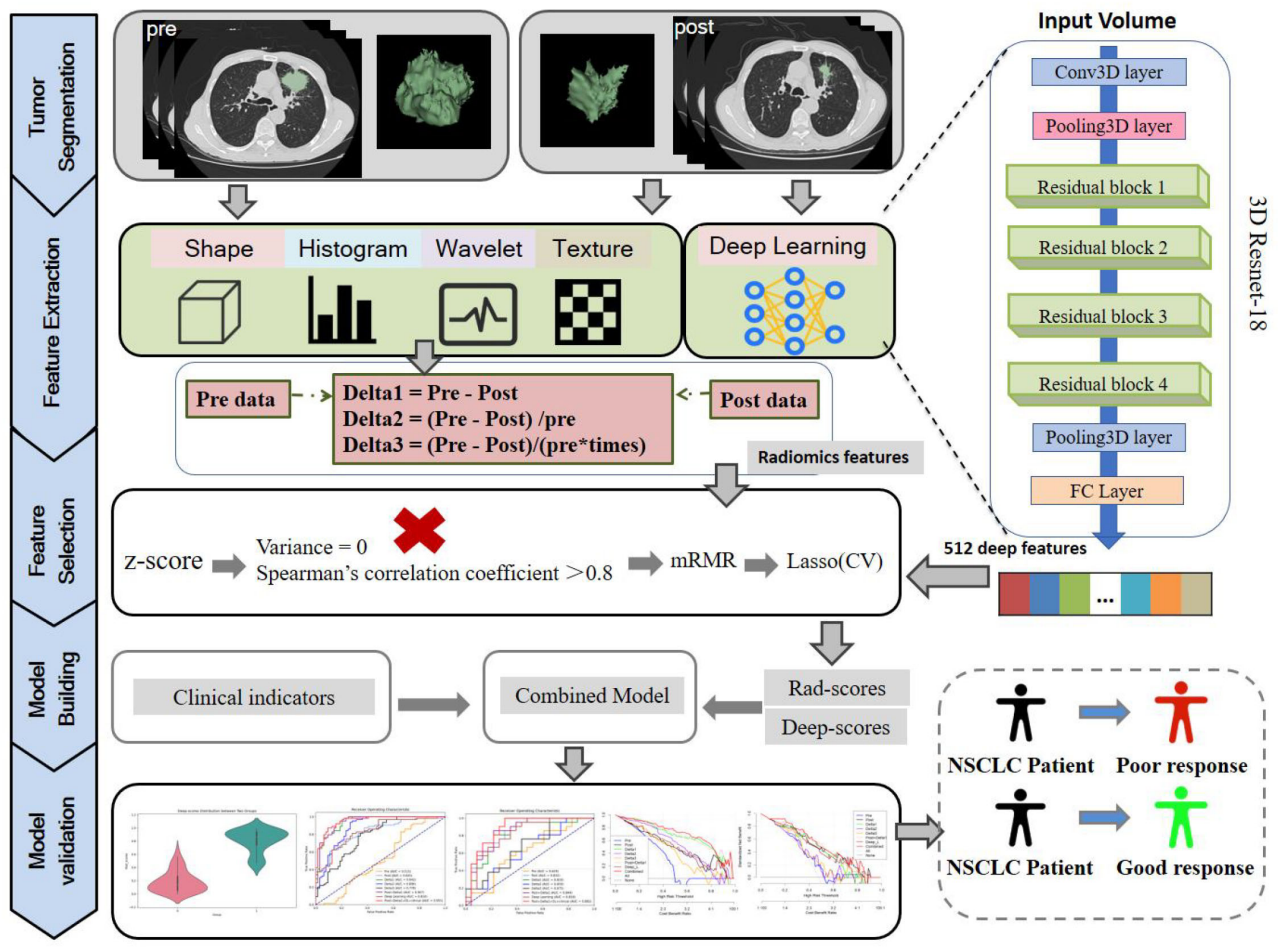
Model construction and assessment flowchart.

Before model construction, the radiomics features from five groups (pre, post, delta1, delta2, delta3) and one deep learning feature group were standardized to ensure comparability across all feature sets. Due to the data imbalance, the Synthetic Minority Over-sampling Technique (SMOTE) was applied to balance the pCR and non-pCR groups at a 1:1 ratio. Crucially, SMOTE was applied only to the training set after the data was partitioned into training and testing cohorts. All testing sets—including both internal and external validation cohorts—consisted solely of original, real-world data, ensuring no data leakage occurred. To reduce collinearity, features with zero variance and those with Spearman correlation coefficients exceeding 0.8 were excluded. The Max-Relevance and Min-Redundancy (mRMR) algorithm was then employed to rank and select the top 30 predictive features, ensuring a balance between relevance and redundancy. This selection corresponds to 3%–10% of the total features, as supported by references (22, 23). Subsequently, the Least Absolute Shrinkage and Selection Operator (LASSO) regression was used to further refine the feature set by identifying the most significant predictive features. Finally, a 5-fold cross-validation was conducted to fine-tune the model parameters and determine the optimal λ value. A logistic regression classifier was then built using the selected features to predict the pathological response (pCR or non-pCR). Model construction and assessment flowchart is displayed in **Figure 2**.

### 2.7 Clinical deep-radiomics combined model construction

The probability predicted from radiomics features was referred to as the Rad-scores, and from DL features as the Deep-scores. The Rad-scores, Deep-scores and clinical variables were identified through univariate and multivariate analyses, and further combined to form a logistic regression model. Model parameters were optimized using 5-fold cross-validation.

### 2.8 Statistical analysis

Statistical analyses were conducted using SPSS (version 23.0) and R (version 4.3.1). Continuous data with skewed distributions were summarized as medians with interquartile ranges (IQR), and group comparisons for these variables were performed using the rank-sum test. Categorical data were presented as frequencies, and group differences were evaluated using the chi-square test or Fisher’s exact test, as appropriate. Single-factor difference analysis test was two-tailed, with a significance level set at α = 0.10. Multivariate analysis was performed using logistic regression. Multivariate statistical test was two-tailed, with a significance level set at α = 0.05. Model performance was assessed by generating Receiver Operating Characteristic (ROC) curves and calculating the area under the ROC curve (AUC). The optimal cutoff value, determined by the Youden index of the ROC curve, was used to compute key performance metrics including accuracy, sensitivity, specificity, positive predictive value, and the F1 score. Additionally, Decision Curve Analysis (DCA) was employed to further evaluate the model’s predictive performance and clinical utility.

## 3 Results

### 3.1 Patient characteristics and groups

Ultimately, 295 patients with NSCLC were collected, and 178 patients were enrolled in this study (Zhejiang Cancer Hospital, 146 patients; Shaoxing People’s Hospital, 18 patients; Huzhou Central Hospital, 14 patients). The whole population had a mean age of 64.39 ± 7.74 years, with 163 (91.60%) patients being male.

Patients were divided into training (102 patients), internal testing (44 patients), and external testing (32 patients) groups. pCR rates were 37.3%, 47.7%, and 34.4% in the respective cohorts. No significant differences were noted between the training and internal testing groups, but significant differences (*p* < 0.05) were found between the internal and external testing cohorts for age, CYFRA21-1, NSE, CEA, and CA199. Significant differences were also found between the training and the external testing cohorts for age, pathological type, CYFRA21-1, NSE, CEA, and CA199. (detailed in **Table 1**)

**Table 1:** Patient and disease characteristics.

Variable		Training cohort (n=102)	Internal testing cohort (n=44)	External testing cohort (n=32)	All patients (n=178)	*p*1	*p*2	*p*3
pCR	Yes	38 (37.3)	21(47.7)	11(34.4)	70(39.3)			
	No	64(62.7)	23(52.3)	21(65.6)	108(60.7)	0.237	0.244	0.768
ICI type	PD-1	93 (91.2)	44 (100.0)	32(100.0)	169 (94.9)			
	PD-L1	9(8.8)	0 (0)	0 (0)	9(5.1)	0.058	/	0.114
Age (years)	mean (sd)	63.17 (7.87)	64.07 (7.54)	68.72 (6.10)	64.39 (7.74)	0.567	0.008^**^	<0.001^***^
Number of treatment cycles	2	74 (72.6)	35 (79.5)	20 (62.5)	129 (72.5)			
	3	19 (18.6)	4 (9.1)	8 (25.0)	31 (17.4)			
	4	9 (8.8)	5 (11.4)	4 (12.5)	18 (10.1)	0.475	0.148	0.281
Sex	Male	91 (89.2)	40 (90.9)	32 (100.0)	163 (91.6)			
	Female	11 (10.8)	4 (9.1)	0 (0)	15 (8.4)	0.900	0.134	0.066
Smoking	Yes	80 (78.4)	39 (88.6)	24 (75.0)	143 (80.3)			
	No	22 (21.6)	5 (11.4)	8 (25.0)	35 (19.7)	0.145	0.119	0.685
Pathological type	squamous cell carcinoma	73 (71.6)	34 (77.3)	30 (93.8)	137 (77.0)			
	adenocarcinoma	23 (22.5)	9 (20.4)	2 (6.2)	34 (19.1)			
	other	6 (5.9)	1 (2.3)	0 (0)	7 (3.9)	0.596	0.137	0.029^*^
Clinical staging	IB	4 (3.9)	2 (4.5)	0 (0)	6 (3.4)			
	IIA	6 (5.9)	2 (4.5)	0 (0)	8 (4.5)			
	IIB	21 (20.6)	8 (18.2)	9 (28.1)	38 (21.3)			
	IIIA	44 (43.1)	20 (45.5)	12 (37.5)	76 (42.7)			
	IIIB	24 (23.5)	12 (27.3)	11(34.4)	47(26.2)			
	IIIC	3 (2.9)	0 (0)	0 (0)	3 (1.7)	0.956	0.496	0.436
iRECIST	iCR	0 (0)	0 (0)	0 (0)	0 (0)			
	iPR	68 (66.6)	36 (81.8)	22 (68.8)	126 (70.8)			
	iSD	32 (31.4)	8 (18.2)	9 (28.1)	49 (27.5)			
	iUPD	2 (2.0)	0 (0.0)	1 (3.1)	3 (1.7)	0.059	0.171	0.863
Cyfra21-1	median [iqr]	5.96 [3.35, 11.40]	5.68 [3.04, 14.56]	4.23 [1.96, 6.28]	5.29 [3.16, 11.40]	0.749	0.023*	0.010*
NSE	median [iqr]	14.41 [11.65, 18.05]	13.90 [11.68, 19.50]	5.27 [2.62,12.26]	13.40 [10.57, 17.90]	0.991	<0.001***	<0.001***
CEA	median [iqr]	2.04 [1.06,4.01]	2.34 [1.05, 4.11]	4.61 [3.03, 6.63]	2.50 [1.13, 4.54]	0.896	0.001**	<0.001***
SCC	median [iqr]	1.50 [0.80,2.62]	2.10 [0.80, 3.85]	1.53 [0.66,2.63]	1.50 [0.80,2.83]	0.511	0.365	0.638
CA125	median [iqr]	17.15 [10.55,29.20]	16.40 [10.63, 42.83]	16.49 [8.60,25.38]	16.85 [10.55,29.20]	0.939	0.542	0.437
CA199	median [iqr]	12.71 [8.75, 24.23]	14.01 [7.63, 24.05]	7.90 [2.58, 15.26]	12.52 [7.51,22.58]	0.991	0.004**	0.001**

*p*1: Training cohort vs internal testing cohort.

*p*2: External testing cohort vs internal testing cohort.

*p*3: Training cohort vs external testing cohort.

*p<0.05, **p<0.01, ***p<0.001.

### 3.2 Analysis of clinical data

To determine the clinical predictors for pCR, we performed differential analyses and multivariate logistic regression analysis. Only iRECIST had a significant difference between the pCR and non-pCR groups (*p* = 0.007) (**Table 2**).

**Table 2:** Statistical analysis of clinical data in the training cohort.

Variable	*p* (difference analysis)	*p* (multivariate analysis)
ICI type	0.915	
Age (years)	0.097	0.556
Number of treatment cycles	0.072	0.314
Sex	0.006^**^	0.999
Smoking	0.066	0.990
Pathological type	0.213	
Clinical staging	0.284	
Cyfra21-1	0.113	
NSE	0.008^**^	0.800
CEA	0.779	
SCC	0.871	
CA125	0.852	
CA199	0.967	
iRECIST	<0.001^***^	0.007^**^

**p<0.01, ***p<0.001.

### 3.3 Model construction and performance comparison

#### 3.3.1 Conventional radiomics model construction and performance

Feature selection resulted in the retention of 1 feature from the pre-radiomics dataset and 12 features from the post-radiomics dataset. AUCs for the pre-radiomics and post-radiomics models were 0.515 (95% CI: 0.420, 0.614) and 0.825 (95% CI: 0.752, 0.895), respectively. For the internal testing cohort, the AUCs were 0.619 (95% CI: 0.451, 0.781) and 0.832 (95% CI: 0.692, 0.935) respectively. Additional performance metrics, including accuracy, sensitivity, specificity, positive predictive value, and F1 scores, are presented in **Table 3**.

**Table 3:** The performance of different models in the training, internal and external testing cohorts.

Model	Cohort	AUC (95%CI)	ACC	SEN	SPE	PRE	F1 score
pre	training	0.515 (0.420, 0.614)	0.469	0.531	0.406	0.472	0.500
	internal	0.619 (0.451, 0.781)	0.568	0.667	0.478	0.538	0.596
post	training	0.825 (0.752, 0.895)	0.742	0.719	0.766	0.754	0.736
	internal	0.832 (0.692, 0.935)	0.727	0.762	0.696	0.696	0.727
delta1	training	0.942 (0.899, 0.977)	0.836	0.812	0.859	0.852	0.832
	internal	0.810 (0.677, 0.933)	0.750	0.714	0.783	0.750	0.732
delta2	training	0.880 (0.818, 0.934)	0.789	0.812	0.766	0.776	0.794
	internal	0.650 (0.482, 0.811)	0.614	0.762	0.478	0.571	0.653
delta3	training	0.778 (0.695, 0.851)	0.719	0.766	0.672	0.700	0.731
	internal	0.675 (0.502, 0.816)	0.614	0.667	0.565	0.583	0.622
Deep	training	0.832 (0.762, 0.898)	0.727	0.750	0.734	0.738	0.744
	internal	0.818 (0.660, 0.930)	0.742	0.714	0.739	0.714	0.714
Combined 1 (radiomics: post-NIT+delta1)	training	0.939 (0.891, 0.974)	0.863	0.737	0.938	0.875	0.800
	internal	0.849 (0.723, 0.954)	0.705	0.619	0.783	0.722	0.667
Combined 2 (radiomics+deep+clinic)	training	0.955 (0.920, 0.984)	0.867	0.828	0.906	0.898	0.862
	internal	0.882 (0.764, 0.975)	0.750	0.714	0.783	0.750	0.732
	external	0.839 (0.686, 0.955)	0.811	0.750	0.857	0.800	0.774

#### 3.3.2 Delta-radiomics model construction and performance

Delta-radiomics analysis involved feature selection from delta1, delta2, and delta3 datasets. This resulted in the selection of 17, 10, and 9 features respectively. The AUCs for the delta1, delta2, and delta3 models in the training cohort were 0.942, 0.880, and 0.778. For the internal testing cohort, the AUCs were 0.810, 0.650, and 0.675 respectively. Detailed metrics are also provided in **Table 3**.

#### 3.3.3 Combined radiomics model construction and performance

**Figure 3:**
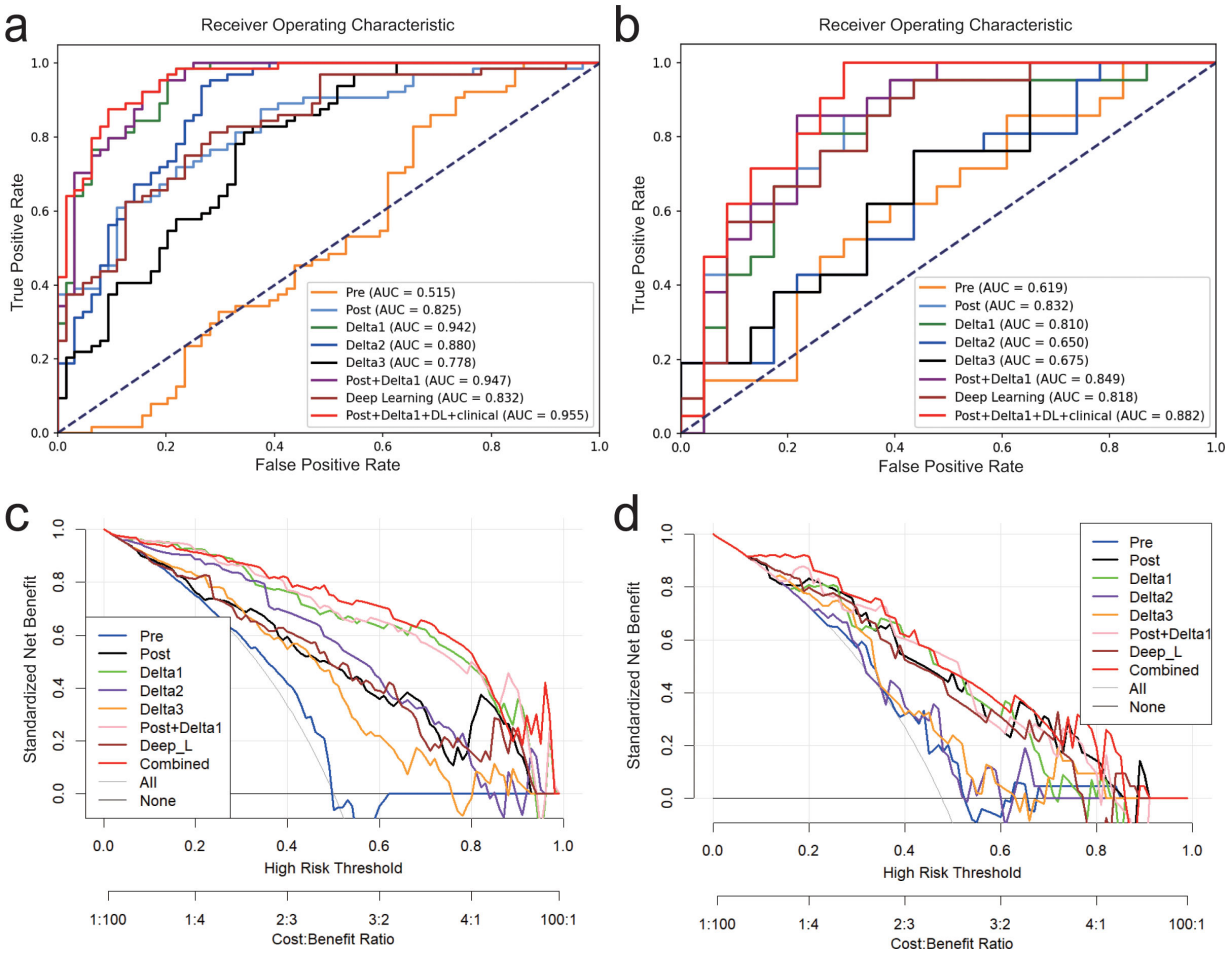
ROC curves of the different models in the training cohort **(a)** and internal testing cohort **(b)**, and DCA for the different models for the training cohort **(c)** and internal testing cohort **(d)**.

Given that the post-radiomics model showed the best performance among conventional radiomics models and the delta1 radiomics model excelled among delta-radiomics models, these two sets of features were combined to develop a more robust predictive model. Nineteen features from two datasets were to form combined model 1 (**Supplementary Figure 1**). The combined model 1 was evaluated using ROC curves (**Figures 3a, b**), which demonstrated AUCs of 0.939, and 0.849 for the training, and internal testing cohorts, respectively. Additional performance metrics are presented in **Table 3**. Furthermore, decision curve analysis are presented in **Figure 3**.

**Figure 4:**
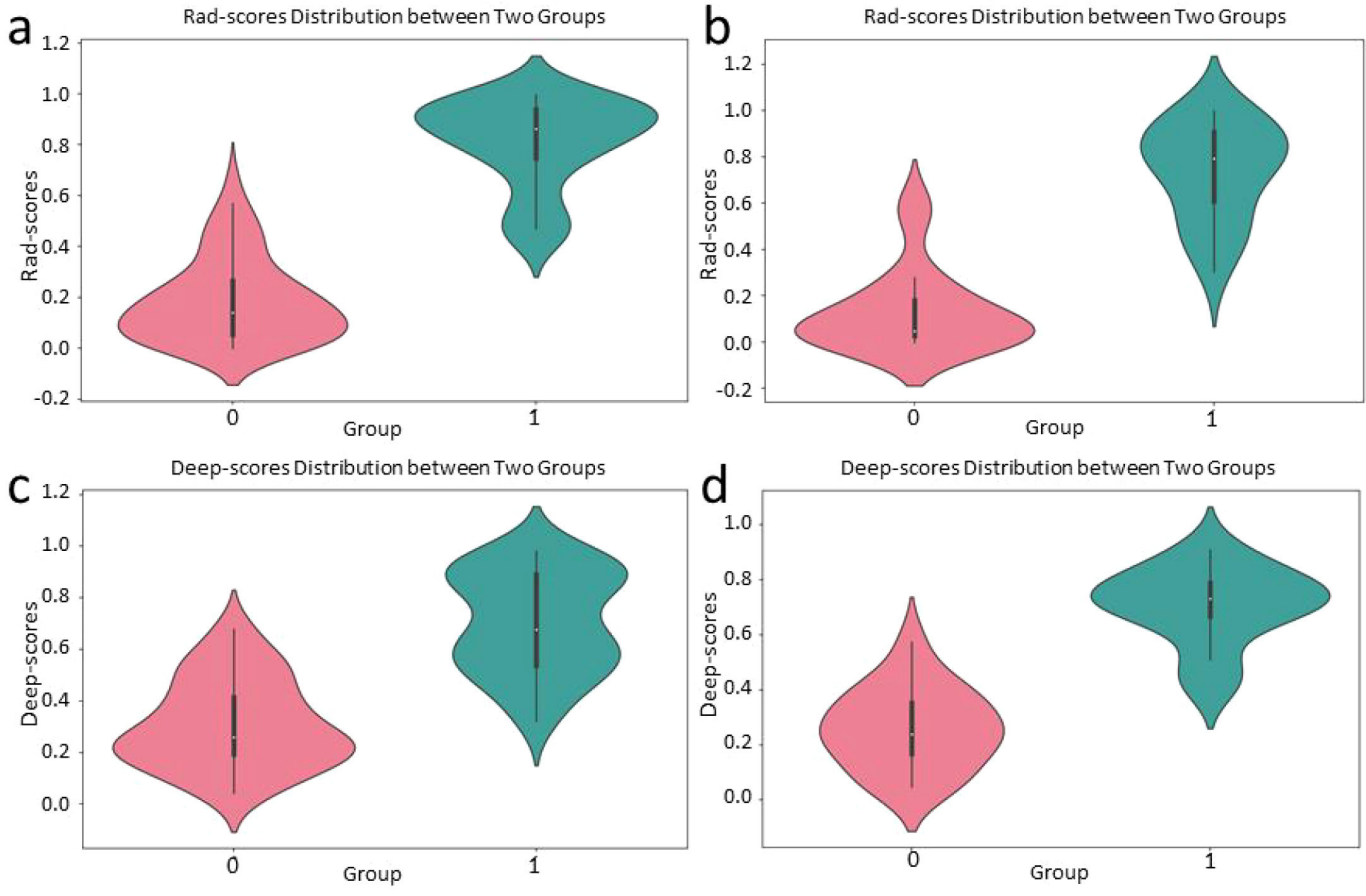
Violin charts of the Rad-scores of the training cohort **(a)** and test cohort **(b)**, and the Deep-scores of the training cohort **(c)** and test cohort **(d)**. The plot highlights a clear separation between the groups, with higher rad-scores and higher deep-scores generally associated with responders (Group 1), indicating the potential of rad-scores and deep-scores as predictive factors for the efficacy of NIT.

The violin plot (**Figure 4**) demonstrates the distribution of rad-scores between the responder and non-responder groups.

#### 3.3.4 Deep learning model construction and performance

After deep learning features extraction, 7 key deep learning features were retained for model construction. A logistic regression model was then built using these selected features, achieving AUCs of 0.832 in the training cohort, and 0.818 in the internal testing cohort. Corresponding accuracy, sensitivity, specificity, positive predictive value, and F1 scores are detailed in **Table 3**.

**Figures 4c, d** depict the distribution of Deep-scores between responder and non-responder groups.

#### 3.3.5 Combined clinical deep-radiomics model construction and performance

The construction of this combined model, referred to as combined model 2, involved the integration of features from the post-NIT radiomics model, the delta1 radiomics model, the deep learning model and iRECIST clinical factor. The combined model 2 demonstrated superior predictive capability compared to the other models. The model achieved AUCs of 0.955, 0.882, and 0.839 for the training, internal testing, and external testing cohorts, respectively. Corresponding performance metrics are detailed in **Table 3**.

### 3.4 Model explanation

**Figure 5:**
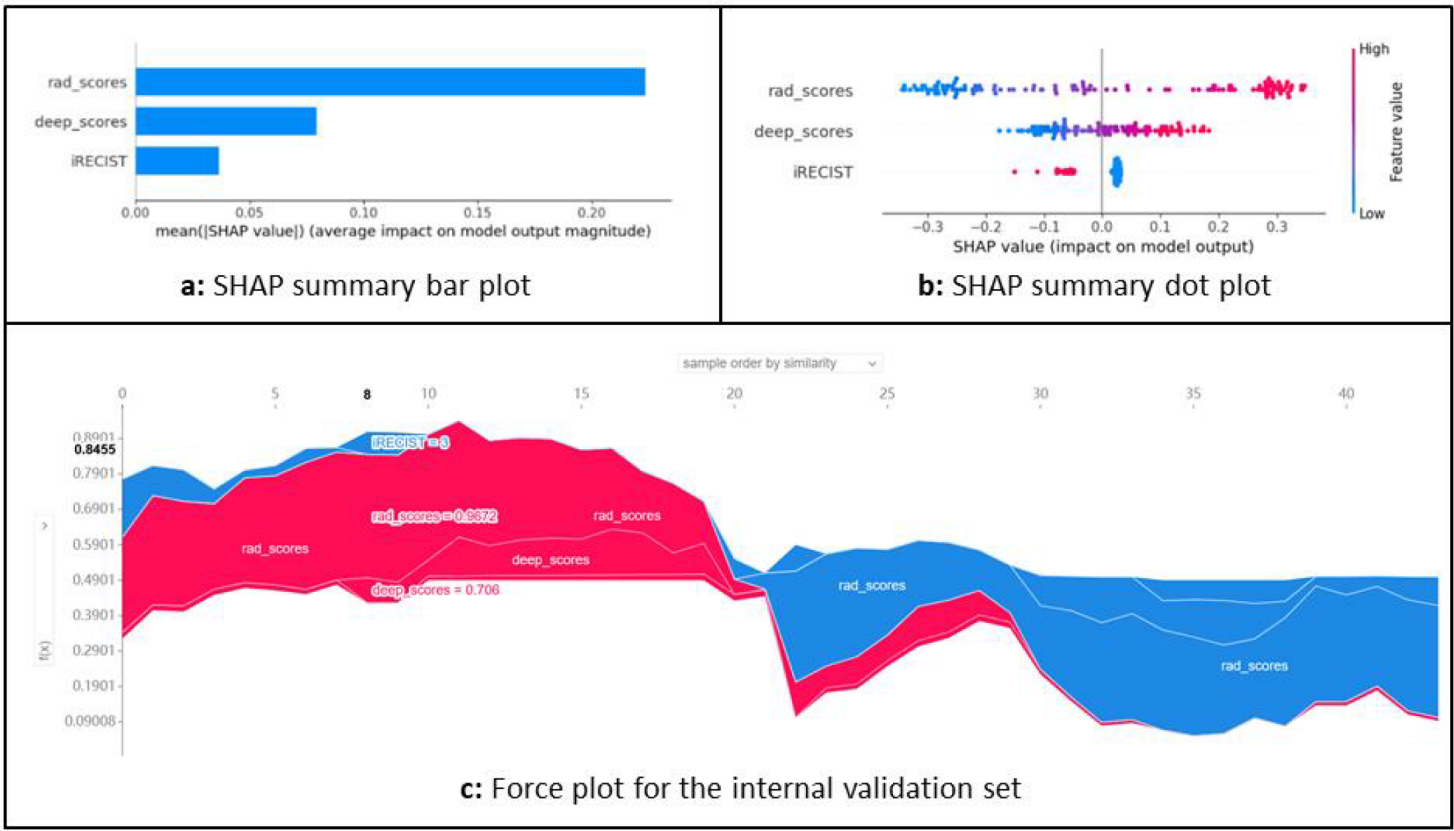
Overall model interpretability using SHAP. **(a)** The SHAP bar plot illustrates the contribution and importance of key features within the combined model. **(b)** The SHAP dot plot demonstrates the direction and magnitude of each feature’s impact on the prediction, with red indicating a positive effect and blue indicating a negative effect on the prediction probability. **(c)** The SHAP force plot provides a comprehensive view of how the Shapley values of each feature influence the prediction for each sample, showing how features contribute to increasing or decreasing the predicted probability relative to the model’s expected output.

As illustrated in **Figure 5**, the SHAP (Shapley Additive exPlanations) method is commonly employed, offering a robust framework for interpreting complex model predictions. SHAP provides both global and local explanations by quantifying the contribution of each feature to the model’s predictions (**Supplementary Results Part 1**).

**Figure 6:**
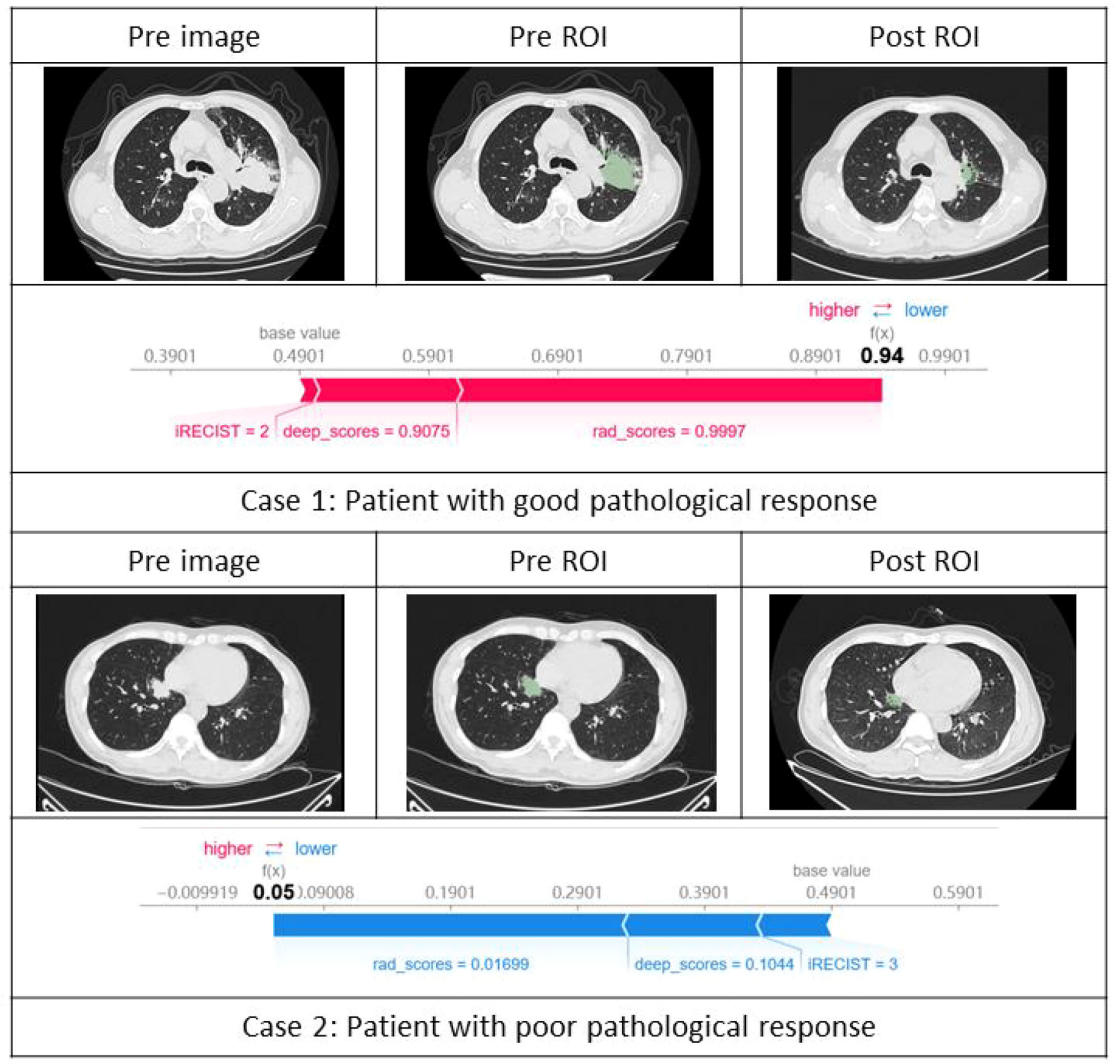
Individual SHAP visualizations. In Case 1, the model’s overall predicted score is 0.94. The SHAP force plot identifies the major contributing features and their specific impact values. Rad-scores is the most significant contributor with a value of 0.9997, followed by deep-scores with a value of 0.9075. The combined influence of these features drives the model to predict a positive pathological response for this patient. In Case 2, the model’s overall predicted score is 0.05. The SHAP force plot indicates that rad-scores (value = 0.01699) and deep-scores (value = 0.1044) are the primary factors leading to the prediction of a negative pathological response for this patient. While the iRECIST feature also shows some influence with a value of 3, its contribution to the prediction is relatively minor.

**Figure 6** shows two typical cases of correctly predicted responder positivity and negativity. These specific SHAP values reveal how the model integrates information from various features to make a decision, highlighting the strong interpretability of the model’s predictions and allowing researchers to better understand the decision-making process.

## 4 Discussion

The current study developed a non-invasive, preoperative tool to predict pCR by integrating clinical parameters, radiomics, and deep learning features derived from CT scans. This model addresses the limitations of traditional models, which are either solely reliant on radiomics features or constrained by the learning capacity of deep learning models when used independently. By merging radiomics, clinical data, and deep learning outputs, the combined model capitalizes on the unique contributions of each data type, and effectively improves the predictive power and robustness in the non-invasive preoperative prediction of pCR in NSCLC patients receiving NIT. The incremental novelty of this work lies in being among the first to synergistically integrate Delta-radiomics with DL features for NIT response prediction. While traditional models often rely on static imaging snapshots, our approach captures both the temporal evolution of tumor heterogeneity (via Delta-radiomics) and high-level semantic morphological transformations (via DL), providing a more comprehensive spatial-temporal characterization of the tumor microenvironment during immunotherapy.

After analyzing the clinical data, serological markers did not exhibit predictive value for treatment response in this study, which contradicts some previous findings. Some studies have shown that high tumor markers before treatment are predictors of poor prognosis. Kataoka et al. (24) reported that high baseline CEA levels were associated with poor progression-free survival (PFS) in advanced NSCLC patients treated with nivolumab. Chai et al. (25) developed a nomogram to predict OS in patients with advanced NSCLC prior to ICI treatment, pointed out CYFRA21–1 level was associated with a shorter OS. Additionally, Huang et al.’s study (26) has shown that the changes in tumor markers can indicate the prognosis of patients, decreases in serological tumor markers (including CEA, CA19-9, CYFRA21-1, and NSE) predicted longer PFS and OS in advanced NSCLC patients receiving single-agent ICI. The discrepancy in our results may be attributed to the fact that the serological indicators included in this study were only the baseline data and the different study endpoints.

Medical images serve as valuable sources of biomarkers, offering a macroscopic view of the tissue of interest (27). Radiomics has the ability to capture intricate details that are undetectable to the naked eye, and its efficacy has been demonstrated in numerous studies (28, 29). Besides, there remain several studies using radiomics to predict NIT in NSCLC patients. Yang et al. (11) constructed PET-based, CT-based, and combined PET-CT radiomics models to predict pCR to NIT in NSCLC, which achieved AUCs of 0.728, 0.732, and 0.818. Notably, the conventional CT-based radiomics model of the current study also demonstrated commendable results, especially the post-radiomics model. This aligns with clinical intuition—post-treatment images directly reflect early treatment-induced tumor changes, which are critical for predicting long-term response. Considering that the changes in the composition of lung cancer after treatment are difficult to be observed by the naked eye, delta-radiomics features were included in this study. Among the three delta-radiomics models, the delta1 model provided an unbiased perspective that reflects absolute difference without any consideration of relativity, and exhibited superior performance. Similarly, Gong et al.’s findings (30) showed that the predictive capability of delta-radiomics model for endpoint surpassed that of baseline (pre-treatment) radiomics model.

From a biological standpoint, rad-scores, derived from radiomics data, could reflect underlying tumor characteristics, such as heterogeneity and vascularization, which has been shown to correlate with treatment response (31). The rad-score in our combined model is determined by both post-treatment conventional radiomics and delta1-radiomics features. These features capture important tumor characteristics before and after treatment, providing a comprehensive evaluation of treatment efficacy. We analyze the most impactful features contributing to the rad-score, focusing on their respective coefficients and how they relate to treatment outcomes. Notably, delta1 features were shown to contribute significantly to predicting treatment outcomes, capturing dynamic changes in tumor characteristics over time. Among the most important delta1 features, delta1-log-sigma-5-mm-3D_firstorder_Minimum (coefficient: 1.4878) represents the minimum intensity value within the region of interest (ROI) after applying a 5-mm logarithmic filter. A post-treatment reduction in this value suggests a decrease in the lowest density regions, potentially indicating necrosis or diminished tumor cellularity, reflective of an effective therapeutic response. Similarly, delta1-wavelet-LLH_firstorder_10Percentile (coefficient: 1.0558), which reflects the 10th percentile of intensity values in the ROI post-wavelet transformation, decreases as low-density regions expand within the tumor, further implying treatment efficacy. Another important feature, delta1-log-sigma-4-mm-3D_ngtdm_Strength (coefficient: 1.0401), measures the smoothness of intensity variations in the tumor, with a post-treatment increase suggesting greater tissue homogeneity, indicative of successful tissue remodeling. In contrast, delta1-wavelet-LHH_glcm_ClusterShade (coefficient: -1.7578), a measure of pixel pair complexity, decreases in post-treatment images, implying reduced textural complexity and cellular heterogeneity, both of which were associated with positive therapeutic outcomes. (more details see in **Supplementary Discussion**) Overall, these radiomics features provide key insights into the tumor’s response to neoadjuvant immunotherapy, offering a quantitative approach for evaluating treatment efficacy and supporting clinical decision-making.

Deep learning has the capability to quantify high-throughput features that are beyond human perception. A previous study (32) suggested that the deep learning score may be associated with pathways involved in tumor proliferation and the enhancement of antitumor immune cell infiltration within the tumor microenvironment. For instance, Trebeschi et al. (33) utilized a deep learning imaging score to predict the prognosis of chemoimmunotherapy in advanced NSCLC, demonstrating that deep learning can capture both tumor- and non-tumor-related morphological changes. Similarly, She et al. (32) developed a deep learning model to predict major pathological response (MPR) in NSCLC patients treated with neoadjuvant ICIs combined with chemotherapy, revealing that higher deep learning scores were associated with an increasing likelihood of achieving MPR. Furthermore, Qu et al. (34) constructed a CT-based deep learning model to predict pCR to neoadjuvant immunotherapy in NSCLC, achieving AUCs of 0.775 in the validation set and 0.743 in the external cohort.

The AUCs of the deep learning model constructed in our study were 0.832 and 0.818 for training and internal testing cohorts, surpassing those of previously reported models mentioned above. As our experimental results showed, patients with high Deep-scores were more likely to achieve pCR. In the context of predicting treatment efficacy, Deep-scores have shown considerable potential in identifying key imaging patterns associated with therapeutic responses. In our predictive models, the inclusion of Deep-scores has significantly enhanced the model’s ability to forecast outcomes. Deep-scores are generated by processing high-dimensional imaging data through convolutional layers in neural networks, which may reflect biological processes like necrosis, fibrosis, or immune cell infiltration, all of which can occur in response to neoadjuvant immunotherapy that may not be easily discerned through traditional radiomics or clinical features (35). However, it is important to acknowledge that these Deep-scores should be interpreted as surrogate imaging patterns that indirectly reflect these biological phenomena rather than direct histopathological evidence. Unlike handcrafted radiomics features, Deep-scores leverage the model’s ability to automatically learn abstract and intricate representations from raw image data, making them especially valuable in capturing subtle variations in tumor structure and behavior over time. Its ability to capture the intricate interplay between different imaging characteristics allows for a more nuanced and accurate prediction of pCR. For instance, a decrease in heterogeneity or a shift in certain Deep-scores may indicate the tumor’s responsiveness to therapy, as regions of viable tumor tissue shrink or become more uniform in texture (36). Moreover, as Deep-scores aggregate multiple layers of imaging information, they may provide a more holistic view of tumor evolution, which can be crucial for distinguishing between responders and non-responders to treatment. Therefore, integrating Deep-scores into treatment efficacy models not only improves predictive performance but also provides a deeper understanding of how specific imaging features correlate with therapeutic success, offering valuable insights for clinicians in treatment planning.

While the results are promising, several limitations in the current study must be acknowledged. The primary limitation of this study is the relatively small sample size, particularly in the external validation cohort. While this may limit the representation of all clinical scenarios, the stable performance across different centers suggests the model’s robustness. We addressed potential overfitting through rigorous feature selection and transfer learning; however, future large-scale studies are necessary to confirm these findings. Additionally, as a retrospective analysis, selection bias is unavoidable, underscoring the need for prospective studies in the future. Moreover, this study focuses on short-term outcomes, and future studies should explore long-term endpoints to provide a more comprehensive understanding of the model’s effectiveness over time.

## 5 Conclusions

In conclusion, our results have demonstrated that the integration of radiomics features, deep learning features, and clinical information could offer superior predictive performance compared to using single-modality information alone. Specifically, the results have shown that the combination of these three sets of features could provide complementary insights, significantly improving the accuracy of pCR prediction in NSCLC patients undergoing NIT. These findings underscore the potential of multimodal integration in clinical predictive modeling and suggest that this approach could serve as the a point-of-care decision tool, enabling personalized treatment planning and ultimately improving clinical outcomes for NSCLC patients.

## Data Availability

The raw data supporting the conclusions of this article will be made available by the authors, without undue reservation.

## Glossary

NSCLC: non–small cell lung cancer
NIT: Neoadjuvant immunochemotherapy
ICI: immune checkpoint inhibitor
PD-L1: programmed death-ligand 1
pCR: pathological complete response
CNNs: convolutional neural networks
CYFRA21-1: cytokeratin 19 fragment
NSE: neuron-specific enolase
CEA: carcinoembryonic antigen
SCC: squamous cell carcinoma antigen
CA125: cancer antigen 125
CA199: cancer antigen 199
iRECIST: immune response evaluation criteria in solid tumors
RECIST: response evaluation criteria in solid tumors
VOI: the volume of interest
ICC: interclass correlation coefficients
SMOTE: synthetic Minority Over-sampling Technique
mRMR: the Max-Relevance and Min-Redundancy
LASSO: the Least Absolute Shrinkage and Selection Operator
ROC: Receiver Operating Characteristic
AUC: the area under the ROC curve
DCA: Decision Curve Analysis
MPR: major pathological response
