# Supplementary material for: The capability of deep-radiomics to predict pathological response to neoadjuvant immunochemotherapy in non–small cell lung cancer: a retrospective multicenter study
Source: Front Immunol. 2026 Feb 27;17:1770042. doi: 10.3389/fimmu.2026.1770042 (PMC12982035; doi:10.3389/fimmu.2026.1770042)
Supplement: Supplementary file 1 [file DataSheet1.docx]

Electronic Supplementary Material

1. **Supplementary Methods**
2. **Supplementary Results**
3. **Supplementary Discussion**
4. **Supplementary Figure**

1. **Supplementary Methods**

Part 1

Initially, the 'Paint' mode was utilized to outline the approximate extent of both the lesion and its surrounding area using distinct colors. Following this, the "Grow from seeds" mode automatically identified both the lesion and its surrounding area more precisely. Finally, the VOI region was manually corrected to avoid major vasculature and the trachea by referencing with the contrast-enhanced sequence.

Part 2

Each dataset consisted of 1,037 radiomics features, which included 107 original features, 186 Laplacian of Gaussian (LoG) features, and 744 wavelet features. The original features comprised 14 shape features, 18 first-order features, and 75 texture features. The texture features were further subdivided into 24 Gray Level Co-occurrence Matrix (GLCM) features, 14 Gray Level Dependence Matrix (GLDM) features, 16 Gray Level Run Length Matrix (GLRLM) features, 16 Gray Level Size Zone Matrix (GLSZM) features, and 5 Neighboring Gray Tone Difference Matrix (NGTDM) features. The LoG and wavelet features were derived from the first-order and texture features. Specifically, LoG features were extracted using LoG filters with sigma (σ) values of 4 and 5, while wavelet features were computed using wavelet transform filters, with eight decompositions performed at each level across the three-dimensional space.

1. **Supplementary Results**

Part 1


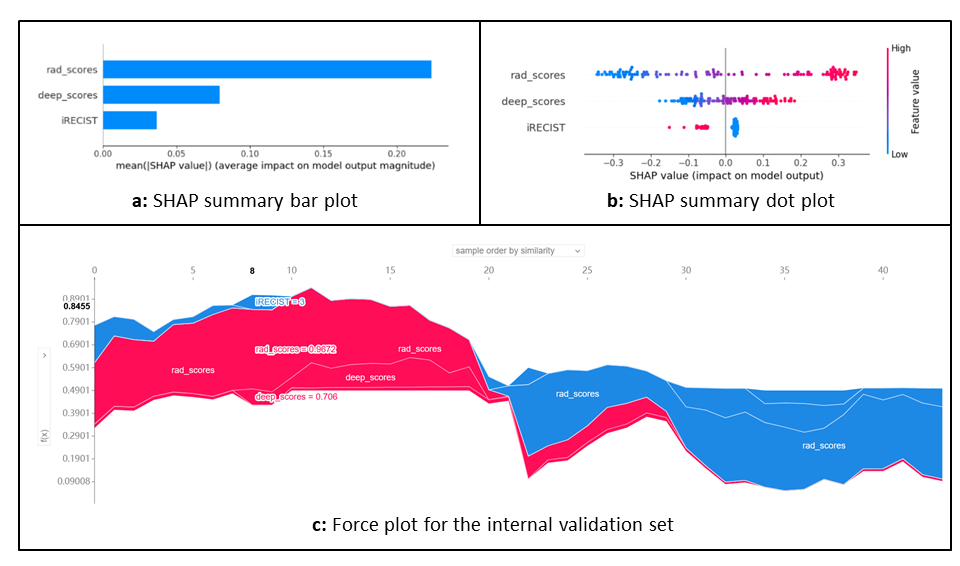


In clinical practice, the adoption of predictive models often hinges on their transparency and interpretability. Clinicians are more likely to trust and implement models whose decision-making processes can be clearly understood. To address this need, the SHAP (Shapley Additive exPlanations) method is commonly employed, offering a robust framework for interpreting complex model predictions. SHAP provides both global and local explanations by quantifying the contribution of each feature to the model’s predictions. The global explanation offers an overview of how the model behaves across the entire dataset, highlighting the most important features driving predictions. On the other hand, the local explanation focuses on individual cases, demonstrating how specific features influence the prediction for each patient. This dual approach ensures that the model remains interpretable and transparent, facilitating its integration into clinical workflows.

As shown in SHAP summary plots (Figure 5a and b), **Figure 5a** presents the SHAP bar chart, summarizing the average magnitude of Shapley values for each feature. This chart quantifies the overall importance of each feature in the model. Rad-scores emerge as the most critical feature, with the highest mean SHAP value, indicating its dominant influence on the model's predictions. Deep-scores and iRECIST, while also contributing to the model, have comparatively lower impacts, as reflected by their smaller mean SHAP values. This bar chart reinforces the findings from the summary plot, underlining the pivotal role of rad-scores in the predictive model. The SHAP dot plot in Figure 5b is a summary plot that visualizes the positive or negative effects of each feature on the output of a machine learning model. The X-axis represents the SHAP values, indicating how much each feature is contributing to the model's prediction; a positive SHAP value means the feature pushes the prediction higher, while a negative SHAP value pushes it lower. The Y-axis lists the features used in the model. Each dot corresponds to a single prediction for a given feature. The color represents the feature's value, ranging from low (blue) to high (red). The dots are stacked vertically to show density. Figure 5b presents the SHAP value distribution for the model's key features: rad-scores, deep-scores, and iRECIST. Each dot represents a sample's SHAP value, with the color indicating the specific feature value (red for high values, blue for low values). It is evident from the plot that rad-scores has the most substantial impact on the model's predictions, with SHAP values spanning a broad range and predominantly on the positive side, indicating a strong positive contribution to the model's output. Importantly, deep-scores also exerts a significant influence on the model's predictions. The SHAP values for deep-scores are widely distributed across samples, and higher feature values are associated with positive shifts in the model's output, underscoring the importance of this feature in the predictive process. In contrast, iRECIST appears to have a minimal effect on the output, with the majority of its SHAP values clustered near zero, though high feature values still contribute slightly to reducing the model's output. Collectively, these results highlight that rad-scores and deep-scores are the primary contributors to the model's predictions, playing critical roles in determining the pathological response. **Figure 5c** shows the SHAP force plot for the internal validation set, which visualizes the distribution of Shapley values for each feature across all samples. The samples are ordered by similarity, revealing distinct clusters where specific features have a dominant impact. In this plot, rad-scores consistently shows a significant positive contribution to predicting the outcome, particularly in a large subset of samples (marked in red). Deep-scores also contribute positively but with smaller magnitude, and their impact is less consistent across the samples. The feature iRECIST exhibits the least influence on the model's prediction, suggesting its relatively minor role in comparison to rad-scores and deep-scores. This visualization effectively highlights how different features contribute differently across various subsets of the data.

1. **Supplementary Discussion**

**Part 1**

Specifically, the post-treatment conventional radiomics features reflect static tumor properties after neoadjuvant immunotherapy, while the delta1-radiomics features quantify dynamic changes in the tumor over time, capturing variations in texture, intensity, and shape that result from treatment. The integration of these two types of features in the rad-score enhances the model’s ability to predict pathological complete response (pCR) with higher accuracy. Below, we analyze the most impactful features contributing to the rad-score, focusing on their respective coefficients and how they relate to treatment outcomes. Among the incorporated features, seventeen delta and two post-treatment features demonstrated distinct and complementary predictive value. Some parts have been discussed in the manuscript. In addition, an increase in delta1-wavelet-HHH_glcm_Idmn (coefficient: 1.1396) suggests a reduction in grayscale variability, reflecting greater tissue uniformity and a favorable response. Lastly, delta1-wavelet-LLL_glcm_ClusterShade (coefficient: 0.9313) indicates changes in tumor texture, where a decrease in complexity reinforces the effectiveness of the treatment. In addition to delta1 features, two post-treatment radiomics features were identified as significant contributors. Firstly, First order_Energy (coefficient: -0.0176), measures the magnitude of voxel intensities, with lower values correlating to a better prognosis, likely due to a reduction in lesion size or density. Secondly, gldm_Large Dependence Low Gray Level Emphasis (coefficient: 0.4968), captures the extent of connected low-intensity regions, correlating positively with a favorable response to therapy, possibly reflecting tumor necrosis or a reduction in viable tumor tissue.

**4. Supplementary Figure**


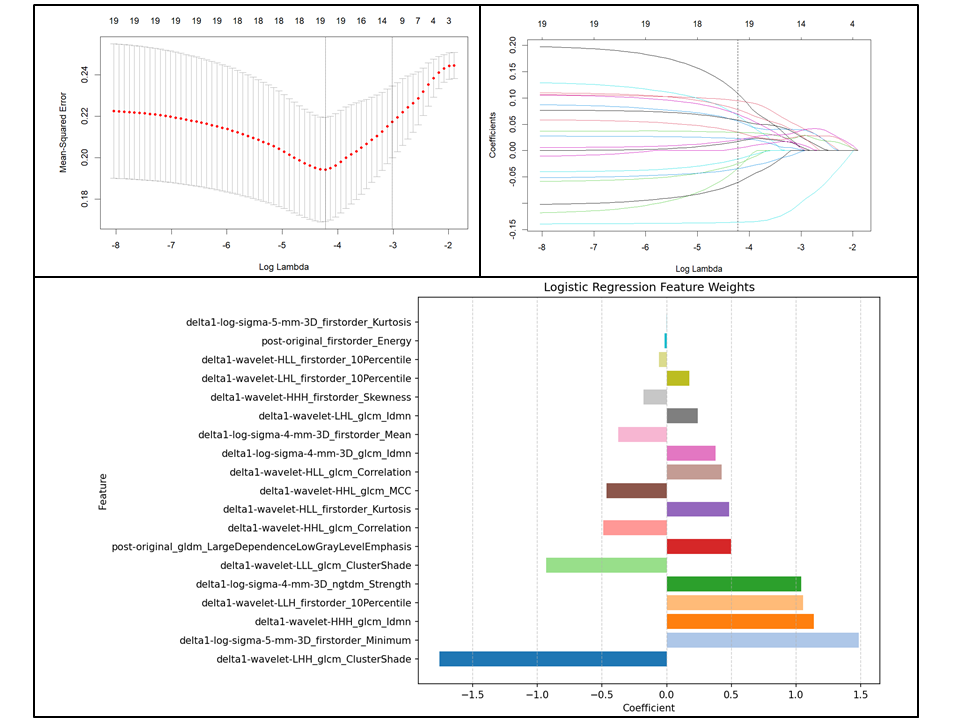


**Figure S1.** The building process of the combined model 1
